# Supplementary material for: New Insights into Potocki-Shaffer Syndrome: Report of Two Novel Cases and Literature Review
Source: Brain Sci. 2020 Oct 28;10(11):788. doi: 10.3390/brainsci10110788 (PMC7693731; doi:10.3390/brainsci10110788)
Supplement: Supplementary file 1 [file brainsci-10-00788-s001.pdf]

Decipher patient 286390

EEG performed during hospitalization because of a status epilepticus secondary to a Haemophilus influenzae septic shock

-04.04.2012: Clinical status epilepticus

Treatment: Rivotril (clonazepam); Diprivan (Propofol); Lamictal (Lamotrigine);

The pattern begins with a rhythmic spike-wave discharge more apparent at the frontal lobe, with episodes of clinical chewing. It has been decided to inject 1mg of Rivotril (at 10:7:53 a.m). The injection appears to be ineffective. Rhythmic spikes still persist, confirming the epileptic condition. A second injection of 1mg of Rivotril (at 10:13:33 a.m.) has been given, which has slightly modified the rhythmic waves, giving a a less jerky appearance, but spikes and waves discharges still persisted, especially on the right side. Status epilepticus. Low effectiveness of the 2 injections of Rivotril.

-05.04.2012: Control

Treatment: Lamictal (lamotrigine), Valium (diazepam), Gardenal (phenobarbital), Rivrotril (clonazepam)

Asymmetric background activity is recorded, at the right hemisphere's expense, which appears slower, wider. The activity in the left hemisphere has a mean frequency of 11 to 12 c/s, amplitude 20μV. There are still interictal critical discharges of an epileptic nature with degraded spikes and waves in the parietal, parieto-occipital and temporo-occipital right regions. The interictal elements are -registered pseudo-periodically every 2 to 3 seconds. The recording shows no evidence of critical rhythmic activity.

-07.04.2012: Control

Treatment: Diprivan (Propofol), Keppra (leviracetam), Lamictal (Lamotrigine)

Asymmetric background activity with straight lateralization signs. The background rhythm is over modulated by medicated fast rhythms and consists of slow waves of 10 to 12 c/s. No recording of spikes or spikes-waves, neither status epilepticus.

-16.04.2012: Control and decrease of Rivotril (Clonazepam) being considered

Traitment: Lamictal (Lamotrigine), Keppra (Leviracetam), Rivotril (Clonazepam)

The pattern starts in standby with a moderately regular, not very active theta activity. We always see some slow waves overloads more specifically in C4-P4. We still note the presence of some fast rhythms in a diffuse way, which can be linked to benzodiazepines. The overall pattern seems to remain not very labile and rather monomorphic. The pattern is quite similar to the previous one (07.04.2012).

First author /Year	ArrayCGH/FISH/Microsatellite	Phenotype	
		Major (cardinal) features	Minor
**1) Decipher patient** ***286390***	**GRCh37/hg19:** 42,333,416-50,378,802	Multiple exostosis<br>Developmental delay<br>Intellectual disability<br>**Epilepsy**	Large abduction and an external rotation of the two hips;<br>Neonatal hypotonia;<br>Thin corpus callosum<br><br>at birth: W=2970g (-0,87 SD); H=51cm (+0,56 SD); FOC=34cm (-0,54 SD);<br>12 years: W=31 kg (- 3 D.S.); H=134 cm (- 2 D.S.); BMI=17 kg/m² (between P25 and P50)
**2) Decipher patient** ***415213***	**GRCh37/hg19:** 45,670,806 45,993,729<br>*de novo*	Global developmental delay<br>Severe intellectual disability<br>**Epileptic encephalopathy-** ***West like***	Café-au-lait spots;<br>Benign external hydrocephalus;<br>Bilateral mastoiditis<br><br>born 4060gr (>85th percentile), length 52cm (85th);<br>at 5 year of age weight 23kg (90-97th percentile), height 121cm (>97th percentile), head circumference 53,5 cm (90-97th percentile)
**3) McCool et al. 2017**	**GRCh37/hg19:** 34,027,915-45,104,910<br>unknown	Multiple exostoses<br>Biparietal foramina<br>History of mild developmental delay	Intermittent strabismus due to congenital right superior oblique muscle palsy;<br>Recurrent infections

1 |Supplementary table 1: position and phenotype

			40 week of gestation 3,175 g (15th <P<50th) and 49 cm (50th)<br>age 4 years 8 months revealed a height and weight at the 96th and 91st centiles and FOC just under the 98th centile
**4) Labonne et al. 2015**	**GRCh37/hg19:** 45,923,612-46,142,729<br>unknown<br><br>after Real Time qPCR correction:<br>1.STARTS: *MAPK8IP1* between first and fourth exon: NM_005456; exon 4: 45,923,531-45,923,612 2. ENDS: *within '5UTR PHF21A*<br>NM_016621; first exon: 46,142,729-46,142,486	Global developmental delay<br>Craniofacial anomalies	Minor limb anomalies; Micropenis; Milled syndactyly; Tapered fingers; Bilateral clinodactyly;<br><br>birth weight of 3.32 kg (25th percentile), a length of 53 cm (75–91th percentile), and an occipitofrontal head circumference of 34.5 cm (25th percentile);<br>22 months, the patient had a weight of 17.28 kg (99.6th percentile), a height of 89.3 cm (98th percentile), and a head circumference of 50.6 cm (75th percentile)
**5) Sohn et al. 2014**	**GRCh37/hg19:** 39,204,770-47,791,278<br>unknown	Multiple exostosis<br>Biparietal foramina<br>Developmental delay<br>Severe intellectual disability<br>Dysmorphic face	Hypotonia; Ptosis; Nystagmus; Strabismus<br><br>after 40 weeks of gestation with birth weight of 2,400 g (< 3rd percentile);<br>at age 6 yr, height was 96 cm (<3rd percentile), weight was 15.7 kg (<3rd percentile), and head circumference was 45 cm (<3rd percentile)

**6) Kim et al. 2012**<br>***GC14361***	**GRCh37/hg19:**33,473,554-47,320,004<br>unknown	Bilateral foramina<br>Midline parietal cortical defect<br>Developmental delay<br>Dysmorphic features<br>Encephalopathy	Hypotonia; Sensoneural hearing loss; Unilateral absent testis; Recurrent otitis media; Slight pectus excavatum; Slender fingers; Small phallus (micropenis); Short stature
**7) Montgomery et al. 2012**	**GRCh37/hg19:** 45,833,960-45,970,465<br>*de novo*	Global developmental delay<br>Subtitle dysmorphic futures	Hypotonia;<br>Mild pectus excavatum;<br>Bilateral pes planus<br><br>39 weeks gestation with a birth weight of 4,366 g (95th centile) and birth length of 53 cm (90th centile).<br>at 32 months, height, weight, and head circumference measurements plotted at the 95th–97th centiles.
**8) Palka et al. 2012**	**GRCh37/hg19:** 44,017,269-46,343,049<br>unknown	Developmental delay<br>Multiple Exostoses<br>Severe mental retardation<br>Facial dimorphism<br>**Epilepsy**	Birth weight, length and head circumference values within normal parameters
**9) Romeike et al. 2007**	**GRCh37/hg19**:38,868,079-50,682,194<br>unknown<br>*position from Kim et al.[1]	Parietal foramen<br>Palpable bony defects in the parietal bones<br>Global developmental delay<br>Intellectual disability<br>**Seizures**	Hypertrophic obstructive cardiomyopathy; Generalized brain atrophy with large ventricles; Focal dysplasia of the cerebellar cortex; Thin corpus callosum; Choroid plexus cholesteatoma; Multiple osteochondromas; Contractures of the

			knees and osteoporosis; Undescended testis; Anemia and thrombocytopenia; *Deceased from septic toxic cardiac arrest*
**10) Bremond et al. 2005**			
Patient 1 (WAGR+PSS)	**GRCh37/hg19:**30,038,577-44,205,103<br>unknown; FISH<br><br>*D11S151- RP11-104M24<br>*additional position from Crolla et al.[2]<br>CO8160 (D11S151, *KCNA4*)<br>*KCNA4*:30,031,288-30,038,577<br>RP11-104M24: 44,205,103-44,357,631	Multiple exostosis<br>Facial dysmorphism<br>Mental retardation associated with mood disorders	Bilateral aniridia; Left kidney tumor; Ptosis; Horizontal nystagmus; Cataract; Diffuse corneal opacities; Bilateral aphakia; Ocular hypertension; Severe truncal obesity;<br><br>When examined at age 25, the patient was 150cm tall (-2.4 SD), weighted 95 kg (+7 SD) and had an OFC of 55 cm (median).
**11) Chuang et al. 2005**			
Patient 1 Interstitial deletion 11(p11.12p.11.2)	**GRCh37/hg19:** 45,030,472-45,952,091<br>maternal; microsatellite analysis<br><br>D11S554- D11S1319<br>D11S554:44,830,234-45,030,472<br>D11S1319:45,952,091-46,152,450	Biaprietal foramina<br>Global developmental delay<br>Craniofacial abnormalities	Micropenis; Esotropia
Patient 2: brother 7y	Not available	Biaprietal foramina<br>Multiple exostosis<br>Mental retardation<br>Febrile seizures and **Epilepsy**	Umbilical hernia
Patient 3: brother 12y	Not available	Biaprietal foramina<br>Mental retardation<br>Febrile seizures and **Epilepsy**	Cryptorchidism
**12) Wakui et al. 2005**			

PSS03	**GRCh37/hg19:**42,922,228-44,166,825<br>paternal; FISH/microsatellite markers<br><br>D11S1355-D11S3805<br>D11S1355:42921915-42,922,228<br>D11S3805:44,166,825-44166957	Biaprietal foramina<br>Multiple exostosis<br>Dismorphic features	Osteochondroma; Bowing of the lower extremities; Short stature; Brachydactyly<br><br>at 4 years height, weight, and head circumference were <3rd, <3rd, and 50th percentiles, respectively
PSS04	**GRCh37/hg19:**44,110,035**-**48,353,905<br>paternal; FISH/microsatellite markers<br><br>D11S1121-D11S1180<br>D11S1121:44109749-44,110,035<br>D11S1180:48,353,905-48354265	Biaprietal foramina<br>Multiple exostosis<br>Developmental delay<br>Dismorphic features	Hypotonia; Myopia; Umbilical hernia; Bilateral inguinal hernia; Hypospadias; Micropenis; Multiple upper respiratory tract infections and otitis media; Hypogammaglobinemia; Brachydactyly<br><br>at 3 years, 10 months his height, weight, and FOC were at the 10th, 60th, and <5th percentiles, respectively
PSS08	**GRCh37/hg19**:31,052,844-46,248,029<br>paternal<br>*position from Kim et al.[1]	Biprietal foramina<br>Developmental delay<br>Dysmorphic features	Wilms tumor; Aniridia;<br>Agenesis of corpus callosum
PSS10	**GRCh37/hg19:**40,405,682-46,977,776<br>paternal; FISH/microsatellite markers<br><br>D11S4455- D11S1085<br>D11S4455:40405393-40,405,682<br>D11S1085:46,977,776-46978119	Biaprietal foramina<br>Multiple exostosis<br>Severe speech delay<br>Dysmorphic features	Strabismus; Hypoplastic corpus callosum; Short hands and feet; Micropenis<br>*Deceased from multiorgan failure*<br><br>at 11 years height, weight, and FOC were at 75th, 75th, and 90th percentiles, respectively.

PSS12	**GRCh37/hg19:**42,922,228-47,133,045<br>paternal; FISH/microsatellite markers<br><br>D11S1355-D11S2039<br>D11S1355:42921915- 42,922,228<br>D11S2039:47,133,045- 47133358	Parietal foramina<br>Developmental delay<br>Dismorphic features	Fingertip anterior fontanel;<br>Agenesis of corpus callosum;<br>Bilateral choroid plexus cysts;<br>Mild to moderate sensorineural hearing loss; Small testis.<br><br>appropriate for gestational age<br>at 13 months, his weight, length, and head circumference were at the 90th, 90th, and 40th percentiles, respectively
PSS13	**GRCh37/hg19:**41,871,599-51,382,783<br>paternal; FISH/microsatellite markers<br><br>D11S1779-D11S1395<br>D11S1779:41871194-41,871,599<br>D11S1395:51,382,783-51383036	Biparietal foramina<br>Global developmental delay<br>Craniofacial anomalies	Hypotonia; Cryptorchidism; Visual impairment with esotropia; Hearing impairment; Ventricular septal defect;<br>Mildly widened CSF spaces<br>History of multiple otitis media and multiple respiratory infection;<br>Brachydactyly; Short stature<br><br>intrauterine growth retardation;<br>at age 35 months the patient was below the 5th percentile for head circumference, weight, and length
**13) Wuyts et al. 2004**			
Patient 1	**GRCh37/hg19:**43,995,113-49,324,458<br>*de novo;* FISH/microsatellite analysis<br><br>D11S1393- D11S1326<br>D11S1393:43,994,800-43,995,113<br>D11S1326:49,324,458-49,324,874	Biaprietal foramina<br>Multiple exostosis<br>Facial dimorphisms<br>Developmental delay<br>Extreme delay of	Hypotonia; High myopia; Markedly hypoplasia on the cerebellar vermis/ hemispheres; Obesity

		speech development<br>Intellectual disability<br>**Seizure**	normal with birth weight 3000 g (P50) and length 52 cm (P90)<br>at age 21 years her height 160 cm (25<P<50) and her weight 70 kg (P90). Occipital-frontal circumference 54 cm (25<P<50)
Patient 2	**GRCh37/hg19:** 42,922,228-44,930,234<br>*de novo;* FISH/microsatellite analysis<br><br>D11S1355- D11S554<br>D11S1355:42,921,915-42,922,228<br>D11S554:44,930,234-44,930,472	Multiple exostosis<br>Developmental delay<br>Intellectual disability	Hypotonia; Anal atresia; Anal fistula; Ventrally placed anus; Ventricular septal defect; Horizontal nystagmus; Convergent strabismus; Syndactyly; Small hands; Tapering figures<br><br>birth weight and length were 3280 g (P90) and 51cm (P75)<br>age 7 years and 2 months, she was 120 cm tall (25<P<50) and her weight was 23 kg (P50). Head circumference was 48.4 cm (<P3)
Patient 3<br>***described by Bartsch 1996* [3] *and Lorenz 1990* [4]**	**GRCh37/hg19:**40,974,567-49,324,458<br>*de novo;* FISH/microsatellite analysis<br><br>D11S905- D11S1326<br>D11S905:40974273-40,974,567<br>D11S1326:49,324,458-49324874	Biaprietal foramina<br>Multiple exostosis<br>Facial dysmorphisms<br>Developmental delay with autistic behavior<br>Intellectual disability<br>**Seizures**	Hypotonia; Myopia; Nystagmus; Strabismus; Blepharophimosis; Meningoencephaloceles; Adipose aspect; Micropenis; Tapering fingers, Simian crease; Acrocephalosyndactyly; Mild cutaneous syndactyly of fingers 2-5; Cone-shaped epiphyses of the middle phalanges; Small hands;

			born at term (birth weight 2,950 g, length 49 cm);<br>at 14 years height was approximately 143cm (<P3), weight 25 kg (<P5), and head circumference 50cm (<P3).
**14) Chien et al. 2003**<br>***three family members***	**GRCh37/hg19:**41,871,599-49,324,458<br>maternal; FISH/microsatellite markers<br><br>*position from Wakui et al, patient PSS07 [5]<br>D11S1779-D11S1326<br>D11S1779:41871194-41,871,599<br>D11S1326:49,324,458-49324874	Bilateral parietal foramina<br>Developmental delay<br>Abnormal craniofacial appearance<br><br>*mother's brothers suffered from febrile seizures **epilepsy** and had intellectual disability	Hypotonia; Esotropia; Mild ventriculomegaly; Thin corpus callosum; Micropenis;<br><br>* mother's brothers<br>the 12-year-old brother had right-side cryptorchidism, whereas the 7-year-old brother umbilical hernia.<br>their CT revealed thin corpus callosum and mild ventriculomegaly.
**15) Hall et al. 2001**			
Patient 7626	**GRCh37/hg19:**43,878,160-45,063,096<br>maternal; FISH/microsatellite markers<br><br>*position from Wakui et al, patient PSS05 [5]<br>RH13886-SHGC-85351<br>RH13886:43877855-43,878,160<br>SHGC-85351:45,063,096-45063446	Parietal Foramina<br>Multiple Exsostosis	
Patient 7625	**GRCh37/hg19:**43,878,160-45,063,096<br>paternal; FISH/microsatellite markers<br><br>*position from Wakui et al, [5]<br>RH13886-SHGC-85351	Biaprietal foramina<br>Multiple exostosis	Asthma;<br>Short stature;<br>Hyperactivity

	RH13886:43877855-43,878,160<br>SHGC-85351:45,063,096-45063446		
Patient 7628	**GRCh37/hg19:**43,878,160-45,063,096<br>paternal; FISH/microsatellite markers<br><br>*position from Wakui et al, [5]<br>RH13886-SHGC-85351<br>RH13886:43877855-43,878,160<br>SHGC-85351:45,063,096-45063446	Biaprietal foramina;<br>Multiple exostosis	
**16) Wuyts et al. 1999**			
Four family members	**GRCh37/hg19:**44,146,056-44,278,811<br>paternal; FISH/microsatellite markers<br><br>D11S903-D11S2095<br>D11S903:44,145,910 44,146,056<br>D11S2095:44,278,811-44278923	Enlarged parietal foramina (Catlin mark)<br>Multiple exostosis<br>**Seizures** (only in the proband)	Mild hypoplasia of the occipital lobe, vermis, and cerebellar hemispheres<br>*brain CT performed only in two patients
**17) Bartsch et al. 1996**			
Patient 2	**GRCh37/hg19:**36,023,482-46,166,860<br>unknown; microsatellite analysis<br><br>D11S935-D11S1344<br>D11S935:36023164- 36,023,482<br>D11S1344:46,166,860- 46167226	Mulitple exostoses<br>Severe mental retardation<br>Mild facial dysmorphism<br>**Focal seizures developed later into epilepsy**	Marked muscular hypotonia;<br>Strabismus; Nystagmus;<br>Adipose appirance; Micropenis;<br>Tapered fingers<br><br>birth and postnatal growth parameters (weight, length, and occipitofrontal circumference) within normal limits.
Patient 6-8<br>***6-proband***	**GRCh37/hg19:**44,146,056-44,930,234<br>unknown; microsatellite analysis	6/7/8- Multiple exostosis<br>Parietal foramina	*6* – Cafe-au-lait spots on the trunk;<br>Capillary hemangioma on the left

7-maternal grandmother ***8-maternal aunt***	D11S903-D11S554 D11S903:44145910-44,146,056 D11S554:44,930,234-44930472		shoulder; Mild bilateral fifth finger clinodactyly; History of self-abusive behavior and hyperactivity; *8*-Adipose appearence <br>*6*- at birth appropriate for gestational age; At age of 4 years heights is at the 50th centile, weight is at the 10th centile, and head circumference is at the 10th centile.
**18) Potocky et al. 1996**	**GRCh37/hg19:**40,477,655-46,085,228 paternal *position from Kim et al, PSS02 [1]	Multiple exostosis Parietal foramina Mental retardation Mild global developmental delay Craniofacial anomalies	Alternating esotropia; Bilateral ptosis; Short stature <br>at 9 years of age her height, weight, and head circumference were at the both, 60th, and 80th centiles, respectively.
**19) McGaughran et al. 1995** ***(WAGR+PSS)***	Not avalable	Multiple exostosis Parietal foramina Global developmental delay Mental retardation	Bilateral aniridia; Wilms tumor; Glomerulonephritis; Bilateral simian crease; Lens Opacities; Glaucoma; Poor vision; Hypospadias; Undescended testes; Micropenis; Triradiate pelvis; Platyspondyly; Hypogammaglobinemia; <br>weighed 3700 g at birth at term;

			26 years: height is 150cm (<3rd centile) and his weight is 70 kg (75th centile).
**20) Shaffer et al. 1993**<br>***III-1***<br>***prband***	**GRCh37/hg19:**44,110,035-48,353,905<br>maternal; FISH/microsatellite markers<br><br>*position from Wakui et al, patient PSS01 [5]<br>D11S1121-D11S1180<br>D11S1121:44109749-44,110,035<br>D11S1180:48,353,905- 48354265	Multiple exostosis<br>Biparietal foramina<br>Severe mental retardation<br>Craniofacial dysostosis<br>**Epilepsy**<br>Autistic features	Hypotonia; Borderline hypotiroidisam; (elevated TSH and low normal T4); Delayed/failed puberty; ''Awkward,'' ataxic gait; Choroid plexus cyst; Prominent CSF spaces; Several anatomic variants of the cerebral arteriovenous system; Microcytic anemia; Estropia; Myopia; Heterotropia; Simian crease; Leg-length discrepancy and osteopenia; Sleep disturbances; Brachydactyly;<br><br>Small for gestational age;<br>fifth centile for height, weight, and head circumference throughout childhood.
III-2<br>***proband's brother***	**GRCh37/hg19:**44,110,035-48,353,905<br>maternal; FISH/microsatellite markers<br><br>*position from Wakui et al, [5]<br>D11S1121-D11S1180<br>D11S1121:44109749-44,110,035<br>D11S1180:48,353,905- 48354265	Multiple exostosis<br>Severe mental retardation<br>Craniofacial dysostosis	Hypotonia<br>Low normal T4, borderline high TSH;

II-4<br>***maternal half uncle***	**GRCh37/hg19:**44,110,035-48,353,905<br>unknown; FISH/microsatellite markers<br><br>*position from Wakui et al, [5]<br>D11S1121-D11S1180<br>D11S1121:44109749-44,110,035<br>D11S1180:48,353,905- 48354265	Multiple exostosis<br>Biparietal foramina<br>Severe mental retardation<br>Aggressive and self-injurious behavior<br>Craniofacial dysostosis<br>**Seizures**	Hypotonia; Micropenis; Cryptorchidism; Madelung deformity; Sensorineural hearing loss; Strabismus; Mild-to-moderate myopia; Simian crease; Mega cisterna magna or a posterior fossa cyst resulting in mild cerebellar hypoplasia; Adipose appearance

Note: ArrayCGH-comparative genomic hybridization; FISH- Fluorescence in situ hybridization; FOC- Frontal-Occipital Circumference;
Legend: blue: growth parameters ≥90th percentile; brown: growth parameters ≤10th percentile; violet: WAGR symptoms

(1) Kim, H. G.; Kim, H. T.; Leach, N. T.; Lan, F.; Ullmann, R.; Silahtaroglu, A.; Kurth, I.; Nowka, A.; Seong, I. S.; Shen, Y.; Talkowski, M. E.; Ruderfer, D.; Lee, J. H.; Glotzbach, C.; Ha, K.; Kjaergaard, S.; Levin, A. V.; Romeike, B. F.; Kleefstra, T.; Bartsch, O.; Elsea, S. H.; Jabs, E. W.; MacDonald, M. E.; Harris, D. J.; Quade, B. J.; Ropers, H. H.; Shaffer, L. G.; Kutsche, K.; Layman, L. C.; Tommerup, N.; Kalscheuer, V. M.; Shi, Y.; Morton, C. C.; Kim, C. H.; Gusella, J. F. Translocations Disrupting PHF21A in the Potocki-Shaffer-Syndrome Region Are Associated with Intellectual Disability and Craniofacial Anomalies. *Am. J. Hum. Genet.* **2012**, *91* (1), 56–72. https://doi.org/10.1016/j.ajhg.2012.05.005.

(2) Crolla, J. A.; Van Heyningen, V. Frequent Chromosome Aberrations Revealed by Molecular Cytogenetic Studies in Patients with Aniridia. *Am. J. Hum. Genet.* **2002**, *71* (5), 1138–1149. https://doi.org/10.1086/344396.

(3) Oliver Bartsch, Wim Wuyts, Wim Van Hul, Jacqueline T. Hecht, P. M.; Debra Hogue, Walter Werner, Bernard Zabel, Georg K. Hinkel, C. M. P.; Lisa G. Shaffer, and P. J. W. Delineation of a Contiguous Gene Syndrome With Multiple Exostoses, Enlarged Parietal Foramina, Craniofacial Dysostosis, and Mental Retardation, Caused by Deletions in the Short Arm of Chromosome 11. *Am. J. Hum. Genet.* **1996**, No. 58, 734–732.

(4) Lorenz, P.; Rupprecht, E.; Tellkamp, H. An Unusual Type of Acrocephalosyndactyly with Bilateral Parietooccipital "Encephalocele", Micropenis, and Severe Mental Retardation. *Am. J. Med. Genet.* **1990**, *36* (3), 265–268. https://doi.org/10.1002/ajmg.1320360302.

(5) Wakui, K.; Gregato, G.; Ballif, B. C.; Glotzbach, C. D.; Bailey, K. A.; Kuo, P. L.; Sue, W. C.; Sheffield, L. J.; Irons, M.; Gomez, E. G.; Hecht, J. T.; Potocki, L.; Shaffer, L. G. Construction of a Natural Panel of 11p11.2 Deletions and Further Delineation of the Critical Region Involved in Potocki-Shaffer Syndrome. *Eur. J. Hum. Genet.* **2005**, *13* (5), 528–540. https://doi.org/10.1038/sj.ejhg.5201366.

Table 2. Summary statistics of the clinical presentation

**Clinical presentation**	***n***	**%**	**PSS**	***ALX/EXT4***	***PHF21A***
Developmental delay	22	55	17	2	3
Intellectual disability	21	53	17	2	2
Language delay	3	8	3	0	0
Hypotonia	16	40	14	1	1
Epilepsy	14	35	12	1	1
Corpus callosum	8	20	8	0	0
Prominent CSF spaces	6	15	6	0	0
Other brain anomalies	9	23	6	2	1
Micropenis	9	31	8	0	1
Cryptorchidism	8	28	8	0	0
Cataract	2	5	1	1	0
Strabismus	12	30	10	2	0
Nystagmus	5	13	4	1	0
Hearing loss	4	10	4	0	0
**TOTAL**	40				

First author	Dysmorphic features	
	characteristic	additional
**1) Decipher patient 286390**	high and broad forehead, sparse lateral eyebrows, long nose with prominent nasal bridge, short and smooth philtrum, thin lips, short neck	prominent chin with horizontal crease, large mouth
**2) Decipher patient 415213**		mild turricephaly
**3) McCool et al. 2017**	broad forehead, sparse eyebrows	triangular face, mid-facial hypoplasia, narrowed nasal bridge, mild retrognathia
**4) Labonne et al. 2015**	brachycephaly, broad forehead, mild epicanthic folds, broad flat nasal bridge with a full nasal tip, large posteriorly rotated ears, full cheeks, mild micrognathia, small mouth with a thin upper lip	scaphocephaly, slightly prominent metopic ridge, mild malar hypoplasia
**5) Sohn et al. 2014**	microcephaly, sparse eyebrows, prominent nasal bridge, hypoplastic nare, down turned mouth, high arched palate	
**6) Kim et al. 2012** ***GM14361***	microcephaly, epicanthal fold, micrognathia, small mouth	short forehead, flat midface, flat occiput, bulbous nasal tip, depressed nasal root, protuberant ears
**7) Montgomery et al. 2012**	partial right epicanthal fold, short philtrum	thin upper lip vermilion, faint philtral groove
**8) Palka et al. 2012**	high forehead, laterally sparse eyebrows, hypoplastic alae nasi, thin lips, short philtrum	upslanting palpebral fissures

**9) Romeike et al. 2007**	microcephaly, brachycephaly, broad forehead	hypotelorism abnormal position of the eyelids, thin palpebral fissures, dysplastic low set ears, very thin lips, long narrow nose, hypoplastic mandible
**10) Bremond et al. 2005**		
Patient 1 (WAGR+PSS)	micrognathia	downslanted palpebral fissures, low set ears, patulous lips, flat malar areas, crowded teeth
**11) Chuang et al. 2005**		
Patient 1	brachycephaly, downturned mouth angle	frontal bossing, mild upslanting palpebral fissures, low set ears with mild helix dysplasia
**12) Wakui et al. 2005**		
PSS03	brachycephaly, short philtrum	macrocephaly
PSS04	brachycephaly, microcephaly, sparse lateral eyebrows, epicanatal folds, prominent nasal bridge, prominent nose, short philtrum, downturned mouth	protuberant ears
PSS08	brachycephaly, sparse lateral eyebrows, epicanthal fold, prominent nasal bridge, prominent nose, downturned mouth	
PSS10	brachycephaly, epicanthal fold, prominent nasal bridge, downturned mouth	short palpebral fissures, deep set eyes
PSS12	brachycephaly, broad forehead, sparse lateral eyebrows, prominent nasal bridge,	small nose

	hypoplasia of the nares, short philtrum, downturned mouth	
PSS13	microcephaly, brachycephaly, sparse lateral eyebrows, broad forehead, prominent nasal bridge, hypoplastic alae nase, short philtrum, downturned mouth	polygonocephaly, prominent nose
**13) Wuyts et al. 2004**		
Patient 1	brachycephaly, high forehead	turricephaly, large ears, left facial asymmetry, small flat nose, narrow maxilla, prominent high chin
Patient 2	brachycephaly, high and broad forehead, epicanthus, hypoplastic alar wings, upslant of palpebral fissures,	prominent glabella, medially prominent forehead, occiput appeared flat, temporal narrowing, large ears, narrow pointed nose, full cheeks, prominent chin, small teeth, diastemata between maxillar and manibular incisive
Patient 3 ***Described by Bartsch 1996 and*** *Lorenz 1990*	brachycephaly, broad and high forehead, small upturned nose with broad tip, broad nasal bridge, hypoplastic alae nasi, downturned mouth corners, epicanthus, short nec	mild turricephaly, hypertelorism, antimongoloid eye slants, telecanthus
**14) Chien et al. 2003**	down-turned mouth;	frontal bossing; low set ears; mild Mongolian eyeslants (upslanting palpebral fissures), mild helix dysplasia
**17) Bartsch et al. 1996**		

Patient 2	brachycephaly, high and broad forehead, broad nose tip, downturned mouth, short neck	slight mongoloid (upslanting) eye slants, small nose
Patient 6-8	6-high forehead	
**18) Potocki et al. 1996**	brachycephaly, epicanthal folds, prominent nasal bridge, short philtrum, downturned upper lip	
**19) McGaughran et al. 1995**	broad and short neck	coarse facies, buphthalmus, posteriorly angulated low set ears
**20) Shaffer et al. 1993**<br>***III-1***<br>***proband***	brachymicrocephaly, epicanthus, high and broad forehead, sparse lateral eyebrows, downturned mouth, prominent nasal bridge, prominent nose; short philtrum; broad/depressed nasal tip; micrognathia (mild)	telecanthus
III-2<br>**proband's brother**	broad nasal bridge, epicanthus, brachymicrocephaly,<br>(high and broad forehead, downturned mouth, small upturned nose with a broad tip and hypoplastic alae nasi (additional information from Bartsch et.al) [1]	telecanthus
II-4<br>**maternal half uncle**	brachymicrocephaly, epicanthus, high and broad forehead; broad/depressed nasal tip; prominent nasal bridge; short philtrum; downturned mouth	telecanthus

(1) O Bartsch , W Wuyts, W Van Hul, J T Hecht, P Meinecke, D Hogue, W Werner, B Zabel, G K Hinkel, C M Powell, L G Shaffer, P. J. W. Delineation of a Contiguous Gene Syndrome With Multiple Exostoses, Enlarged Parietal Foramina, Craniofacial

Dysostosis, and Mental Retardation, Caused by Deletions in the Short Arm of Chromosome 11. *Am. J. Hum. Genet.* **1996**, *58* (4), 734–742.
